# Conservation Agriculture Impacts on Economic Profitability and Environmental Performance of Agroecosystems

**DOI:** 10.1007/s00267-023-01874-1

**Published:** 2023-10-16

**Authors:** Lorenza Alexandra Lorenzetti, Andrea Fiorini

**Affiliations:** 1 ALTIS – Alta Scuola Impresa e Società, Università Cattolica del Sacro Cuore, Via Necchi 5/9, 20123 Milano, Italy; 2 Department of Sustainable Crop Production, Università Cattolica del Sacro Cuore, Via Emilia Parmense 84, 29122 Piacenza, Italy

**Keywords:** Climate change, Conservation agriculture, Economic and environmental profitability, SOC, Q1, Q5, O3

## Abstract

The rationale of this study originates from the primary sector’s multiple roles in the global warming issue. Agriculture is reported among the main causes of anthropogenic global warming. At the same time, it is profoundly impacted by climate change and concurrently holds potential as a solution through the sequestration of soil organic carbon (SOC) facilitated by Conservation Agriculture (CA). However, the findings in the literature are controversial on the SOC sequestration capacity and the profitability of CA implementation. Considering the new and old objectives of the sector, this paper tackles the assessment of the actual capabilities of CA to be a viable strategy to pursue the social good of climate change mitigation and concurrently be profitable for farmers. The economic profitability and environmental performance of CA are assessed analysing data from a field experiment in Northern Italy (European temperate area) and identifying the best management practice by means of a data envelopment analysis.

## Introduction

More than 90% of climate scientists identify global climate change to be largely a consequence of carbon dioxide (CO_2_) and other greenhouse gasses (GHG)—measured in CO_2_ equivalents—emitted by human activity (Anderegg et al. 2010; Doran and Zimmerman 2009), also known as the “anthropogenic” origin of the climate problem. Amongst the sectors that contribute to global GHG emissions, the primary sector is the second largest emitter^1^, with a hefty part of emissions originating from conventional modern agriculture^2^. The latter has generated an unprecedented increase in agricultural production during the late twentieth century, that comes at a high price of burdensome negative externalities for the environment and for the sector itself, with strong adverse effects on the quality and functionality of soil, water, air, vegetation, and biodiversity, (Benson and Kundis 2014; Grassini et al. 2013; Rahman and Dunfu 2018; Seneviratne et al. 2012; Ussiri and Lal 2019; Zhang et al. 2017; Zhao et al. 2017), as well as eventually affecting the sustainability, resilience and profitability of farms (Gornall et al. 2010; Schipanski et al. 2016) and putting global food security at risk (Gornall et al. 2010; Lal 2015a).

However, the primary sector is also potentially part of the solution. There are various strategies for mitigating climate change resulting from human activities and the relative increased CO_2_ concentration in the atmosphere. Soil in particular, is recognised as one of the major resources for its capacity for CO_2_ absorption (Bai et al. 2019; Lal 2003; Paustian et al. 2016; Yang et al. 2022; Zomer et al. 2017). Hence, among climate change mitigation startegies are those that implement improved land management practices and result in the increase of SOC sequestration (and in most cases in co-benefits for ecosystems and biodiversity), such as: (i) afforestation and reforestation; (ii) agroforestry and other farming combining woody vegetation with croplands; (iii) targeted conversion of cropland to fallow or of set-aside areas to permanent grassland; (iv) restoration of peatlands and wetlands (European Commission 2021). In this paper we focus on SOC sequestration facilitated by Conservation Agriculture (CA). Conservation Agriculture (CA) is achieved through the implementation of three major principles: no soil disturbance (i.e., no tillage), permanent soil cover, and crop rotation (Kassam et al. 2014), and is proposed as a means of implementing this strategy on agricultural land, calculated as one of the major carbon sinks^3^ (Lal 2004; Minasny et al. 2017; Yang et al. 2022; Zomer et al. 2017). Nonetheless, there is a vast literature on CA and a controversial debate. Indeed, this paper writes into a literature comprising divergent conclusions which lead to uncertainty about the capacity of CA to embody a successful climate change mitigation strategy. This calls for further research as also evidenced by existing papers that emphasise the lack of site-specific knowledge (Derpsch 2008; Giller et al. 2015; Kienzler et al. 2012). Furthermore, the effects of the three major principles inspiring CA, namely no soil disturbance (i.e., no tillage), permanent soil cover, and crop rotation (Kassam et al. 2014) in terms of CO_2_ sequestration, yield, and farm profit are not in most of the controversial studies, to the best of our knowledge, assessed together in field experiments, but only in meta-analyses which, as such, are based on secondary data, often including experiments with only some of the three principles, and lose the sought-for site specificity. What emerges is the need for more context-based approaches, studies in situ, farmer-defined best management practices; the assessment of the actual capabilities of CA to be a viable mitigation strategy to pursue the social good of climate change mitigation and concurrently be profitable for the farmer.

This paper fills this void and tackles all three topics of interest (CO_2_ sequestration, yield and farm profit) additionally highlighting the timing of SOC sequestration and the comparative best management practice (BMP), using primary data from a comparative experiment, that is Conventional Agriculture (CT) versus CA, carried out in Northern Italy (Boselli et al. 2020), namely in the European temperate area. The purpose is to assess the environmental performance (in terms of significance and timing, here narrowed down to the positive impact on the environment by way of CO_2_ sequestration in the soil) and the economic profitability of CA with respect to CT. CA is implemented by means of no tillage in combination with cover cropping and residue maintenance, while CT is implemented by conventional tillage and no cover cropping. The scope of the analysis is to be exemplary, albeit with limitations, of what happens in the field in terms of the three issues, so as to be useful to the primary stakeholders: the agricultural entrepreneur and the policy maker. Our analyses show that CA is a BMP for the farmer and a useful complementary climate change mitigation strategy. The paper is organised as follows: Section “Relevant Literature” reports on the relevant literature, Section “Data and Methodology” introduces the data and methodologies used, displays a CA versus CT SOC significance analysis, a gross profit margin analysis and a data envelopment analysis (DEA), Section “Results” presents the results, Section “Discussion” provides discussion and section 6 concludes; an Appendix contains supplementary data.

## Relevant Literature

The paper that gets closest to the analysis carried out here is Sapkota et al. (2015) where the implementation of CA is analysed in terms of costs, yields and environmental impact, albeit in terms of GHG emissions and not CO_2_ sequestration, and with field experiments in the area of the Indo-Gangetic Plains. This is part of a vast literature on CA, which has ignited a heated debate motivated by the seriousness and urgency of the objectives that are to be achieved through its implementation, and to the inconsistent and diverging empirical evidence related to the success of this strategy.

For sure, the capacity of CA to act as a soil CO_2_ sequestration strategy is not unanimously supported. The proposition of CA as a mitigation strategy (Bai et al. 2019; Kassam et al. 2011; Kassam et al. 2014; Lal 2004, 2020; Smith et al. 2008a, 2008b) is often questioned in meta-analyses that reveal inconsistent CA CO_2_ sequestration results, reporting that conversions from Conventional Tillage (CT) to NoTill (NT) change the distribution of carbon in the soil profile significantly, but do not increase the total Soil Organic Carbon (SOC) content (Govaerts et al. 2009; Luo et al. 2010; Powlson et al. 2016). This inconsistency partly originates in the overlapping of the two terms NT and CA, though the relationship is in fact content-container, and implementing only one principle is not implementing CA (Lal 2015). “True” CA is considered to be practiced only when all three principles are applied (Derpsch et al. 2014). González-Sánchez et al. (2012) consider NT + Cover Crop (CC) in their meta-analysis and report positive results in terms of CO_2_ sequestration; Kiran Kumara et al. (2020) meta-analysis in South Asia found carbon sequestration significantly higher in CA-based management practices compared to CT. Likewise, Govaerts et al. (2009) evaluate that altering crop rotation along with NT can influence CO_2_ sequestration; and Luo et al. (2010) report that compared with CT, NT in double cropping systems significantly increases total soil carbon.

Another issue that emerges from the literature, apparently underlying the heterogeneity of the reported CO_2_ sequestration results, is the depth at which SOC is measured. Baker et al. (2007) and Powlson et al. (2016) bring to the fore what they define as a bias in the sampling protocol, considering that in most studies the measurement occurs in the first 0–15 cm or 0–30 cm and in the studies where sampling extended beyond that depth, conservation tillage showed no consistent accrual of SOC, rather a different redistribution. Luo et al. (2010) present a meta-analysis where measurements beyond 40 cm depth find that CA, compared with CT, significantly increased soil total carbon but only in double cropping systems. However, Zomer et al. (2017) estimate that up to 53% of the 4p1000 Initiative target^4^ could be reached in the top 30 cm of cropland soils alone, and continue for over 20 years at least, thus confirming CA as a climate change mitigation strategy.

An ineludible objective of any agricultural system that is to be alternative to conventional agriculture is food security. Also in this domain, the literature reporting on the results of CA implementation on yields is contradictory. Pittelkow et al. (2015, 2015a) report of a variable impact on crop yields depending on the specific crop, with negative or at most matching yield responses in the first years. They conclude their meta-analyses by saying that overall NT yields were reduced in comparison to CT but, when NT is combined with residual retention and crop rotation, negative impacts are minimised, and rainfed crop productivity in dry climates increases significantly rather, suggesting that it may become an important climate change adaptation strategy for ever-drier regions of the world, but not in humid climates (Pittelkow et al. 2015). Giller et al. (2015) meta-analysis also challenges the claims that CA increases crop yields but concurrently reports comparatively higher stability of crop yields in dry climates. Corbeels et al. (2020) claim that the crop yield benefits that can be expected from CA are relatively small and report that average yields under CA are only slightly higher than those of conventional tillage systems in their African sub-Saharan meta-analysis. Den Putte et al.’s (2010) European meta-analysis concludes that in spite of (limited) negative effects on yields, CA is a viable option for European agriculture from the viewpoint of agricultural productivity, as long as interaction with soil type, crop type and climate are accounted for. Kiran Kumara et al.’s (2020) South Asia meta-analysis documents that CA implementation results in gains in yields. Laxmi et al. (2007) find yields increase in India after NT implementation, particularly consequent to more timely planting, allowing also for a potential increase in cropping intensity and diversity. Knapp and van der Heijden (2018) assess the temporal stability of NT + CC yields and conclude that it does not differ significantly from that of CT. Alternately, there is convergence regarding CA producing the best yield in arid soils (Corbeels et al. 2014, 2020; De Vita et al. 2007; Giller et al. 2021; Pittelkow et al. 2015).

Regarding economic profitability, it is generally accepted that both NT and CA represent lower costs for the farm (Corbeels et al. 2020; Laxmi et al. 2007; Pittelkow et al. 2015a). However, inconsistencies do emerge from the literature when the optimal size of the farm for a profitable implementation of CA is inspected. Pannell et al. (2014) recommend that CA is implemented in larger, better-resourced farms since they can afford longer time horizons and as a result have lower discount rates compared to smallholders who have higher opportunity costs and higher risk aversion relative to investments (in dedicated machinery). Consonantly, Corbeels et al. (2020) and Giller et al. (2021) suggest the adoption of CA on larger mechanised farms, and limited uptake by smallholder farmers in developing countries, where they also find that cost reductions would be proportionately smaller (Powlson et al. 2016). Pittelkow et al. (2015a) also consider adoption of CA is not without difficulties consequent to opportunity costs. Kiran Kumara et al. (2020), instead, claim that the reported lower operational costs make CA particularly suitable for smallholders in developing countries. In accordance with the latter, Laxmi et al. (2007) find that CA is suitable for smallholders, and in any case scale neutral.

## Data and Methodology

The research consists of a three-fold analysis performed on a primary data panel gathered from a field experiment run from 2011 to 2020 (Boselli et al. 2020); and a secondary data panel on crop prices gathered from the local Chamber of Commerce (Camera di Commercio Industria Artigianato e Agricoltura di Bologna, Italy, 2021). The CA and CT experiments were carried out on adjacent land plots within the research centre farm CERZOO (managed by the Università Cattolica del Sacro Cuore), which allowed for the advantage of a controlled environment with all variables of interest taken account of. Thus, the main criteria for comparison (Nemes 2009) such as geographical proximity, physical (i.e. soil composition) similarities, management and cropping type similarity are respected for, and the recurring biases in this type of comparative analysis are overcome.

The field experiment was run from 2011 to 2020 at the CERZOO experimental farm (45° 00′ 18.0′′ N, 9° 42′ 12.7′′ E; 68 m a.s.l.), in Piacenza, Po Valley, Northern Italy. The field study was established as a Randomised Complete Block (RCB) with four replicates (blocks) and four treatments: on one side, conventional tillage (CT) as a Control; on the other side, three treatments with no-till (NT) plus winter cover crops (CC) as possible CA systems. The treatments were in detail the following: (i) CT, (ii) NT plus rye (Secale cereale L.) as a winter CC, (iii) NT plus hairy vetch (Vicia villosa Roth) as a winter CC, and (iv) NT plus a 5-species mixture (rye 55%; hairy vetch 25%; crimson clover (Trifolium incarnatum L.) 8%; Italian rye-grass (Lolium multiflorum Lam.) 8%; and radish (Raphanus sativus L.) 4%) as a winter CC. Full details on soil characteristics and experiment set-up are reported in Boselli et al., (2020).

The cash crop sequence during the experiment was the following: (1) winter wheat (Triticum aestivum subsp. aestivum L.), (2) maize (Zea mays L.), (3) maize, (4) soybean (Glycine max L. Merr.), (5) winter wheat, (6) maize, (7) soybean, (8) winter wheat, and (9) maize. Winter CCs were sown after harvesting the previous main crop when winter wheat was not settled as the subsequent main crop, since its cropping cycle overlaps that of winter cover crops. Nevertheless, since CCs are agroecological tools with a series of both short- and long-term agro-ecosystem functions (e.g., supporting the following crop growth in the subsequent year, but at the same time increasing soil organic matter in the long-term, as well as improving soil porosity after roots decomposition with time, enhancing nutrient cycling, etc.), their legacy effects should be considered as lasting beyond a single cropping season. Both CCs and cash crops were directly drilled on undisturbed soils under the three CA treatments. Instead, the seedbed preparation under the CT treatment consisted of a conventional ploughing at 35-cm depth, and two rotating harrowing at a 15–20-cm depth before seeding. Under CA treatments, the cover crop cycle was terminated in spring by spraying Glyphosate (2.4 L ha^−1^) and, two weeks later, the main crop was directly sown without chopping CC residues. Each plot was 22-m wide and 65-m long (1430 m^2^). All plots were tilled conventionally before the experiment started (2011).

During the 9-year experiment, data on grain yield of crops (Mg ha^−1^), at 14% kernel moisture content, i.e. the moisture reference for the price of crop grains, soil organic Carbon (SOC) storage (Mg ha^−1^), measured at 0–30 cm depth, and operational costs (Euro ha^−1^) were collected for all treatments. In detail, grain yield of crops was determined annually by manually harvesting three representative areas of 10 m^2^ per plot. Dry matter yield was obtained by oven-drying sub-samples at 105 °C until constant weight. Subsequently, grain yield at 14% kernel moisture content was calculated by dividing each dry yield by 0.86.

Soil samples were collected each year after harvesting the cash crop. For each plot, 3 composite soil cores were collected randomly to a depth of 30 cm. Then, soil samples were air-dried, ground and sieved (2 mm mesh) before determination of SOC concentration (Nelson and Sommers 1996). Soil bulk density (BD) was determined in the same sampling period for each year (on an additional set of samples) by dividing the oven-dry weight of each soil portion by its volume. Soil organic stock (Mg ha^−1^) was computed as the product of SOC concentration, BD and the corresponding soil depth (0–30 cm).

Given the data from the experiment, in this paper we assess:

1. The environmental profitability of CA by means of a statistical significance analysis of the difference between NT SOC levels and CT SOC levels related to time, i.e. when the SOC values difference in CT and NT treated soils becomes significant relatively to time, (given that yields with NT + CC are found to be not significantly different from CT; Boselli et al. 2020).
2. The economic profitability of the NT + CC versus CT field management, by means of a Gross Profit Margin analysis.
3. The composite economic and environmental profitability of the NT + CC versus CT field management practices, by means of a Data Envelopment Analysis (DEA).

To achieve the objectives of the study, statistical techniques and methodologies have been employed in the analyses of the data, including descriptive statistics (Appendix A), regression analysis, Gross Marginal Profit Analysis and Data Envelopment Analysis (DEA). Descriptive statistics, empirical models, analysis of gross profit margin and DEA were run on Excel and R computer software.

### Significance Analysis

The rationale of this analysis is the assessment of the timeliness of the impact of the activities implemented to counter climate change - in our case the impact of NT + CC on SOC. Thus, given that Boselli et al. (2020) report positive impacts of NT + CC on SOC in comparison to CT, we want to assess if and after how long the difference in terms of the impact on SOC between the two systems (NT + CC and CT) becomes significant. The study comprised *n* = 3 crops: winter wheat, maize and soybean in NT + CC and CT treated plots in the time frame of 2011–2020 (full description in Boselli et al. 2020).

**Fig. 1:**
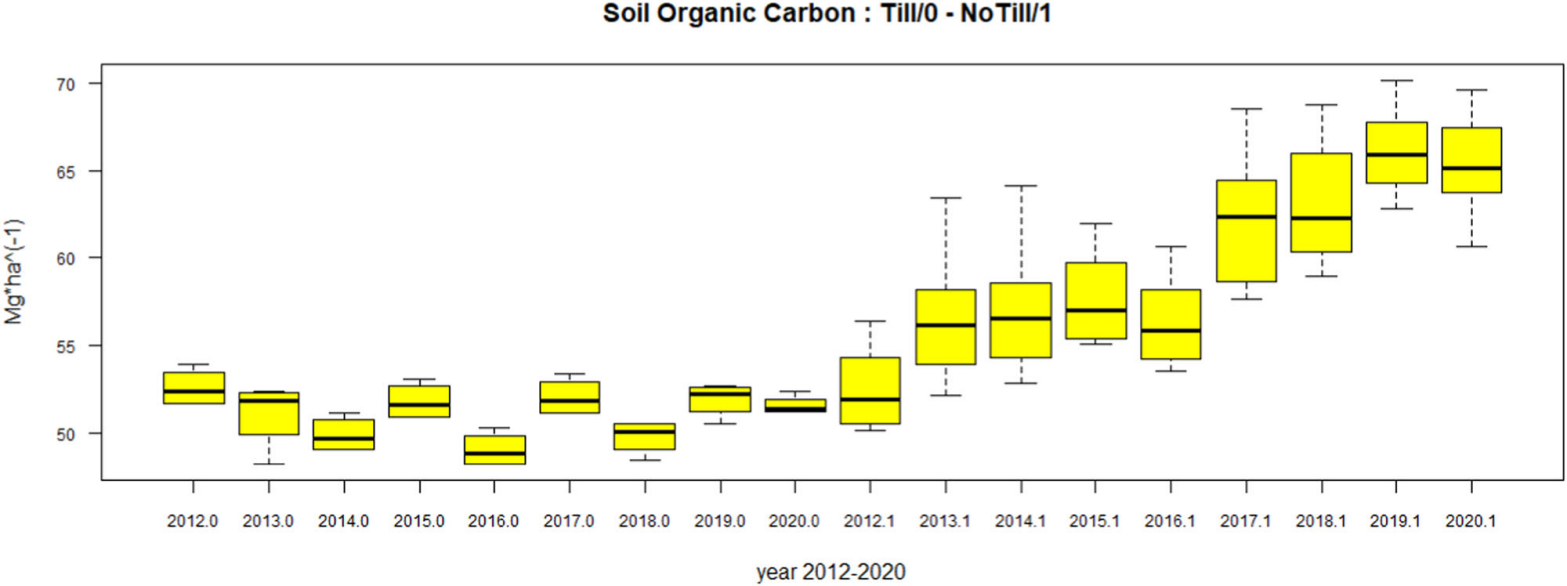
SOC levels in Till (0) and NoTill (1) related to time

A box and whiskers plot (Fig. 1) and a generalised least square (GLS) regression (1), with heteroskedacticity correction, were performed in order to quantify the impact of SOC absorption consequent to the CA field management. The regression model explains the level of SOC as a function of the years from the transition from CT to CA:

1 $$\begin{array}{l}{{{\mathrm{Log}}}}\left( {{{{\mathrm{SOC}}}}} \right)_{{{{\mathrm{i}}}},{{{\mathrm{t}}}}} \,=\, \beta _0 + \beta _1{{{\mathrm{year}}}}_{{{\mathrm{t}}}} + \beta _2{{{\mathrm{NoTill}}}} + \beta _3{{{\mathrm{Soy}}}}_{{{{\mathrm{i}}}},{{{\mathrm{t}}}}}\\\qquad\qquad\qquad +\, \beta _4{{{\mathrm{Wheat}}}}_{{{{\mathrm{i}}}},{{{\mathrm{t}}}}} + \beta _5{{{\mathrm{year}}}}_{{{{\mathrm{i}}}},{{{\mathrm{t}}}}} \times {{{\mathrm{NoTill}}}}_{{{{\mathrm{i}}}},{{{\mathrm{t}}}}} + {{{e}}}_{{{{\mathrm{i}}}},{{{\mathrm{t}}}}}\end{array}$$

In the regression Eq. (1) the dependent variable is the Log(SOC) and the explanatory variables are:

- Year_t_ - the years since the plot has transitioned from T to NT + CC (i.e. from CT to CA),
- NoTill_i,t_ - the dummy variable that distinguishes between a T-treated plot and an NT treated plot,
- Year_i,t_ ·NoTill_i,t_- the slope dummy which captures the impact of the interaction between the years of NT and the crop,
- Main Crop_i,t -_ the control variable, since it is a categorical variable with three levels (soybean, maize, wheat i.e. the type of crop), we used two dummy variables (Soy_i,t_, Wheat_i,t_)
- *e*_i,t_ is the error term.

### Gross Profit Margin Analysis

The rationale of this analysis is that SOC absorption is to date considered a positive externality and at most, a public good without a market. Thus, in the absence of market incentives, the only incentive for a farming business to transition from a conventional to a CA management, which allows for the sought-for SOC absorption, is the relative attainable profitability, if any. Hence, we compare the economic profitability of CT and CA land management by means of a Gross Profit Margin analysis, generally used to determine farm profitability.

While any differences in crop yields and their correlation to SOC levels for the two systems (CT and CA = NT + CC) have been accounted for in Boselli et al. 2020, yields are not per se a characteristic of a production system, and alone do not indicate farm profitability. In fact, farm profitability additionally depends on farm management and production costs. The latter include operational costs (volume related costs) and fixed costs i.e. business costs not dependent on the level of external inputs. Given that the experiment is run on the same farm and that any CA or CT specific hardware/operations, where needed, were subcontracted to third-parties (operational costs are listed in Table A1, Appendix A), fixed costs are the same for CT and CA in this experiment. Thus, we can exclusively consider variable costs in our calculation of the Gross Profit Margins for the conventional agriculture managed plots (CT) and for the CA managed plots (NT + CC).

Moreover, while it occurs that different crop varieties influence the whole rotation (and possibly market prices), this is overcome here since the same varieties are grown both on the CA plots and the CT plots, which are within the same farm and display the same type of soil. We applied average yearly crop prices taken from the local Chamber of Commerce (Camera di Commercio Industria Artigianato e Agricoltura di Bologna, Italy) (Appendix A, Tables A3, A6).

Gross profit margin was calculated as:

2 $${{{\mathrm{Gross}}}}\,{{{\mathrm{profit}}}}\,{{{\mathrm{margin}}}}_{{{i}}} = {{{\mathrm{yield}}}}/{{{\mathrm{ha}}}}_{{{i}}} \times {{{\mathrm{price}}}}/{{{\mathrm{tonne}}}} - {{{\mathrm{operational}}}}\,{{{\mathrm{costs}}}}_{{{i}}}$$

$${{{i}}} = {{{\mathrm{T}}}},{{{\mathrm{NT}}}} + {{{\mathrm{CC}}}}$$

Prices in Euros

### Data Envelopment Analysis (DEA)

Finally, we assess the composite (economic and environmental) efficiency of the CT and NT + CC farm managements. No tillage + cover crop (NT + CC) can be included among technological changes. In fact, it can be defined as a disembodied technological change: it comprises of a different way of combining inputs or using existing resources; it is a change in production technology. This makes it suitable to be measured with the Data Envelopment Analysis (DEA) method (Charnes et al. 1978; Farrell 1957), which is commonly applied to the agricultural sector (Atici and Podinovski 2015).

DEA consists of a nonparametric linear programming model for measuring the efficiency of a decision-making unit (DMU) relative to other DMUs, on the basis of multiple inputs and outputs. The objective of the DEA is to assess the relative efficiency of units which are comparable. The result of this analysis typically brings to the fore a ‘best practice’ frontier. The model has two alternative orientations: input or output DEA (Charnes et al. 1978). For the purposes of this analysis, the Output-oriented DEA is best suited^5^: it singles out the unit that produces the highest level of outputs from a given combination of inputs. Following Roll et al. (1991) the DMUs for the DEA evaluation need to be defined and selected considering the boundaries that affect their determination. These are (1) organisational, physical or regional boundaries which define the individual units, and (2) time related boundaries: the time periods applied in measuring the activities of the DMUs should be “natural”, i.e. corresponding to seasonal cycles, budgeting or auditing periods. In our case the organisational or physical boundaries are overcome since the evaluated DMUs belong to one farm (CERZOO), which avoids potential differences in physical or organisational terms and satisfies the requested homogeneity. Further, though we have a panel data set, we opted for a yearly cross-section analysis, following the natural cycle of crops and the crop change per cycle. We have thus identified four DMUs: CT (conventional agriculture management), NTrye (NoTill + cover crop = rye), NTvetch (NoTill + cover crop= vetch), and NT mix (NoTill + cover crop= mix). These comply to the requested characteristics of being a ‘homogeneous’ set of decision-making units where comparison is sensible. Additionally, we determined the input and output factors which are relevant and suitable for assessing the relative efficiency of the selected DMUs. The input factors must be selected such that they accentuate the basic differences among units. We consider operational costs per DMU as the input factors. The output factors are the gross profit margin, which represents economic profitability, and the level of SOC which represents environmental profitability.

Efficiency is then measured with respect to the DMUs and selected factors, identifying the differences in performance. The chosen performance measures reflect the efficiency of each DMU in terms of economic and environmental profitability. The software used for the calculations is R.

## Results

### Significance Analysis

The impact of NT + CC and Till (T) + crop residue management on the Soil Organic Carbon (SOC) level in the considered time frame (2011–2020) are shown in the Whiskers Boxplot diagram in Fig. 1. The descriptive distribution exhibits the impact of CT (=0 in the figure), on SOC levels as constant in the observed time frame (2011–2020), and the impact of NT + CC (=1 in the figure) on SOC as constantly increasing. In particular, we can observe that after the 3rd year the upper whisker relative to the CT is lower than the lower whisker of the NT + CC.

**Fig. 2:**
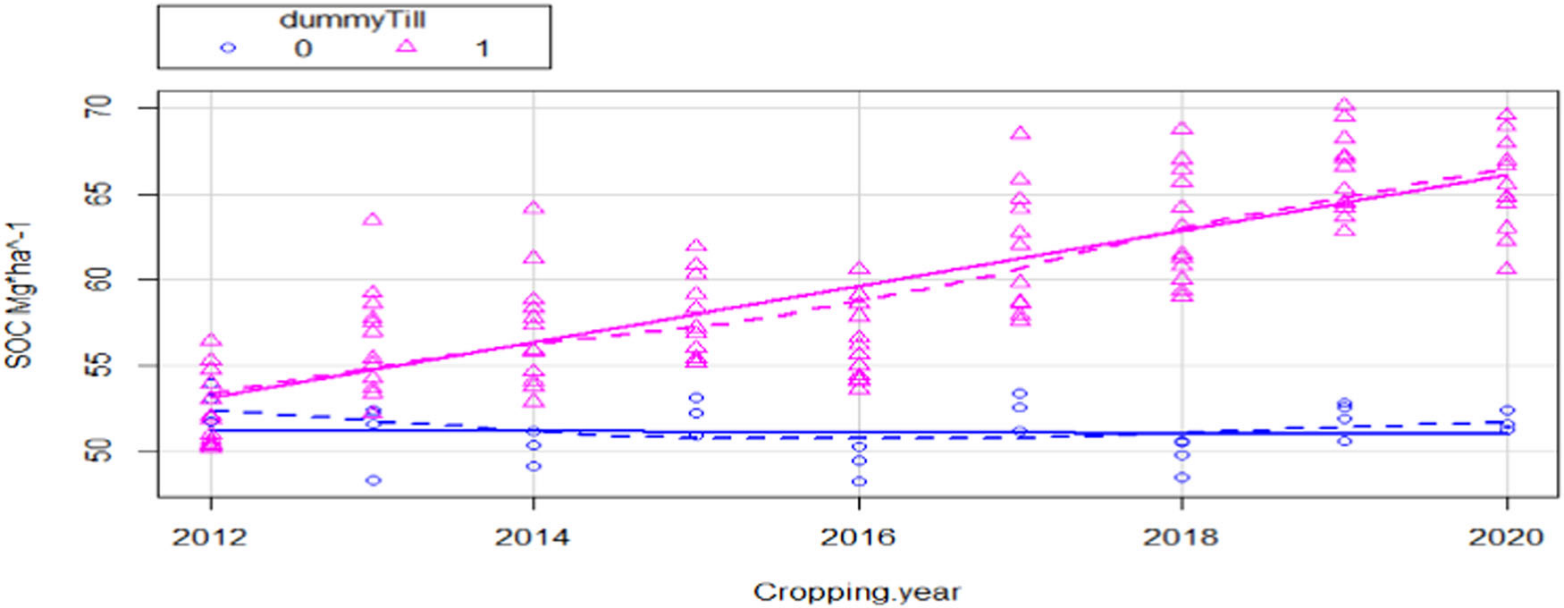
Linear Trend of Till and Aggregated NoTill impact on SOC levels 2011–2020

The GLS regression results (Table 1) highlight a significant improvement of the coefficient of the variable ‘cropping year’ (0.0275) when transitioning from Till to NoTill + CC: the average level of the Log (SOC) increases significantly in the transition from Till to NoTill + CC, all else equal. We verified for the necessary linear regression assumptions: residuals have null mean (*t* test, *p* value = 0.8549), do not deviate from normality (Shapiro test, p value = 0.2122) and do not deviate from homoskedasticity (White test, *p* value = 0.7392). Further, Fig. 2 highlights the linear trend of Till and aggregated NT + CC impact on SOC levels, consequently a comparison of the slope between CT and NT + CC on SOC can be drawn.

**Table 1:** GLS coefficients

	Estimate	Std. error	t value
Intercept	−50.49	0.6158	−81.998***
Cropping.year	0.02708	0.0003	88.697***
Dummy NoTill	55.38	0.8465	65.424***
Main.Crop==Soybean	−0.01128	0.0017	−6.497***
Main.crop==Wheat	−0.01923	0.0015	−13.005***
Cropping.year:dummy NoTill	0.0275	0.0004	−65.628***

Signif. codes: 0 ‘***’ 0.001 ‘**’ 0.01 ‘*’ 0.05 ‘.’ 0.1 ‘ ’ 1

Residual standard error: 1.008 on 138 degrees of freedom

Multiple R-squared: 0.9958, Adjusted R-squared: 0.9956

F-statistic: 6489 on 5 and 138 DF, *p* value: <0.0001

**Fig. 3:**
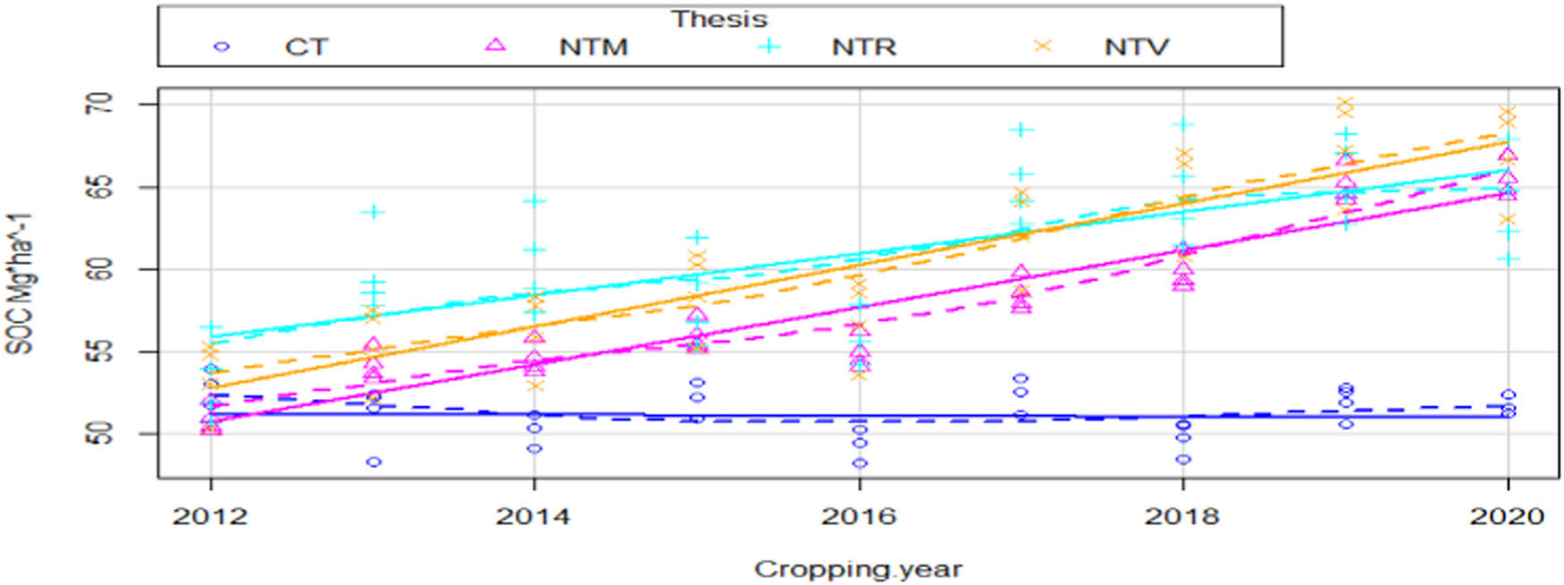
Till (CT) and disaggregated NoTill (NT) + cover crop (Mix, Rye, Vetch) impact on SOC levels

Figure 3 highlights the impact of NT + CC on SOC but differentiating per cover crop (Mix, Rye and Vetch). There is no significant difference in the impact on SOC dependent on cover crop type: the trend is increasing for all three cover crops.

**Fig. 4:**
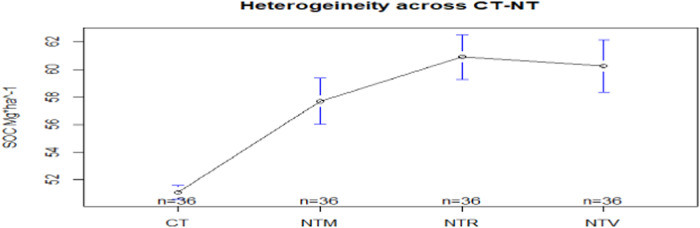
Heterogeneity across NT and T practices on SOC

Figure 4 analyses the variability of SOC levels per NT + CC and CT systems. SOC remains constant in the considered time frame for CT production, while it has a greater variability in NT practices (NTR, NTM, NTV). The levels of SOC are significantly greater under NT + CC. Actually, the lower end of the confidence interval of the average value of SOC for the disaggregated NT + CC is greater than the upper end of the confidence interval of the average value of SOC for the CT. Mean and Standard Deviation for SOC per Crop, and per System (CT CA), are displayed in Table A4, Appendix A.

### Results of the profit margin for CT and RA

**Fig. 5:**
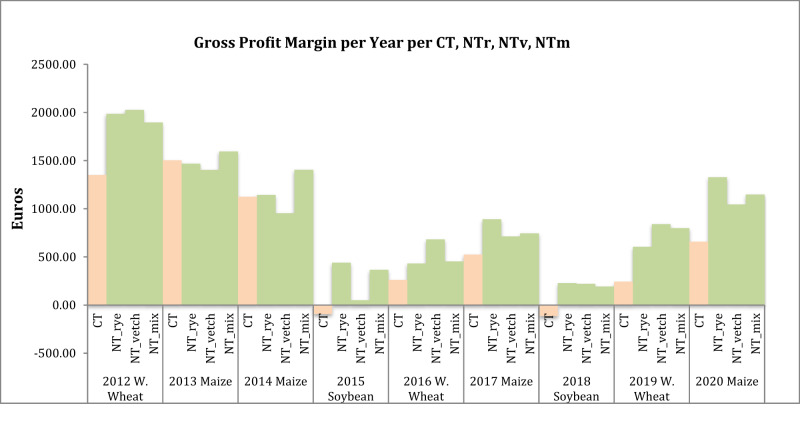
Gross profit margin for conventional tillage and NoTillage per crop, per year

Figure 5 exhibits the results of our Gross Profit Margin analysis per year. The graph highlights that NT + CC managed rotations generally consistently outperform CT managed plots in a range from 6% to 200% in terms of gross profit margin. This is particularly manifest for winter wheat and soy. Only the maize rotation exhibits a slower adaptation to the transition from CT to NT + CC. For the first two maize rotations (2013, 2014), CT management exhibits a higher gross profit margin^6^ but for NT+ mix, which outperforms CT by 6% in 2013 and by 24% in 2014. The displayed impact on the gross profit margin derives exclusively from variations in yields (which result not significant; Tables A2, A5, Appendix A; Boselli et al. 2020) and relative management (NT + CC, CT) operational costs, since all other variables are equal as are the crop varieties grown in the NT and CT plots and hence, their relative selling prices (Table A3, Appendix).

### DEA Results

**Fig. 6:**
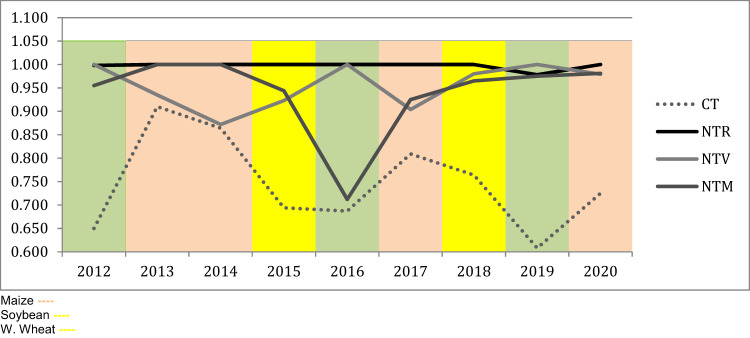
DEA results on composite efficiency (economic & environmental)

Table 2 displays the results of the DEA analysis. At the aggregate level the NT + CC units are more efficient in terms of the assessed composite efficiency. At a disaggregated level, NTrye represents, the frontier of maximum efficiency from which the frontier of values for the CT unit is clearly detached, except for 2019 (i.e. for winter wheat). The least distance between CT and NT efficiency frontiers is displayed for maize, but only in the first two rotations as can also be seen in Fig. 6. Instead, the distance between the CT performance and the NTrye or NTvetch best practice is accentuated for the winter wheat rotation. NT therefore represents an aggregate best practice and cover crops have different impacts depending on the crop, recalling that CA is a site-specific strategy.

**Table 2:**
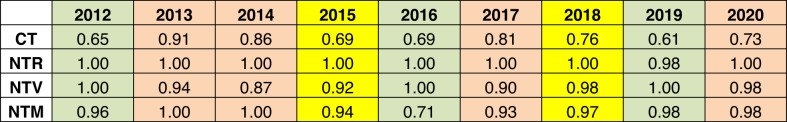
DEA results on composite efficiency (economic & environmental)

*CT* Conventional Till, *NTr* NoTill rye, *NTv* NoTill vetch, *NTm* NoTill mix

Maize

Soybean

W. Wheat

## Discussion

In this paper, we compared Conservation Agriculture (CA = NoTill + Cover Crop) and Conventional Agriculture (CT) land management, both with the same crop rotations, in terms of the timing of Soil Organic Carbon absorption, economic profitability and composite efficiency (environmental and economic). This is accomplished by means of a three-fold analysis - a regression analysis, a gross profit margin analysis and a Data Envelopment analysis on primary panel data from a comparative field experiment in Northern Italy (Boselli et al. 2020). The results of the analyses exhibit a positive comparative response for CA.

Our analysis of soil organic carbon (SOC) cumulation shows that CO_2_ sequestration starts at CA implementation and grows at every cycle. These results refer to measurements in the first 0–30 cm soil and are in line with most results found in the literature. However, it is often considered that the accumulation in this first strip of soil is exclusively the result of a redistribution instead of an accrual in total CO_2_ in the soil; hence, damping if not invalidating the function of CA as a climate change mitigation strategy (Baker et al. 2007; Giller et al. 2021; Govaerts et al. 2009; Luo et al. 2010; Powlson et al. 2016). In fact, Boselli et al. (2020) additionally measure the CO_2_ concentration beyond this depth (in the underlying 30–60 cm strip of soil), where they find equivalence in the SOC between the two systems (CT and CA); concluding that there is comparatively more total SOC for CA treated fields. Given this, our result on the timing of SOC accrual is relevant for manifold reasons. To begin with, the farmer acknowledges a practical example of the timing of CO_2_ absorption in a European temperate zone. Hence, the susceptibility to subsidies from the Common Agricultural Policy (CAP) that would integrate farm profits and potentially impact the farmer’s risk aversion in implementing CA. Furthermore, increasing SOC triggers a virtuous process within the soil, eventually enhancing fertility and productivity (Lal 2002). Calculating the timing of the CO_2_ accrual in the soil is thus relevant also in the latter perspective. Additionally, our results in terms of the CO_2_ accrual timing are better than expected: Derpsch (2008) predicts that measurable differences in CO_2_ content happen only in the transition phase (5–10 years) and are not expected in the initial phase (0–5 years). Our calculations report a significant difference between CT and CA SOC contents from year 3.

For the policy maker, this analysis is an assessment of the effectiveness and relative timing of CA as a complementary climate change mitigation strategy. Together with other strategies for climate change mitigation among which improved land management practices,, the analysis of the timing of CA CO_2_ sequestration is also of interest with respect to the stringent deadlines given by scientists^7^. It consequently provides a rationale for decision-making when comparing with other possible land management practices to fight climate change: reforestation, for example, which has a much slower impact time (40 years) than CA (Bateman and Balmford 2018). The findings are also of interest for decisions related to the establishment of a carbon credit market, in the event that payments are defined relative to a threshold of SOC accrual (in the EU there is an ongoing process of Carbon removal certification definition). Moreover, the analysis concerns an area of the world where the evolution of CA has been comparatively slower but has the second highest total sequestration potential among regions of the world (Derpsch 2008; Yang et al. 2022; Zomer et al. 2017). It concerns Italy which is among the three European areas (along with France and Spain) where CA is considered to have a greater growth potential (Derpsch 2008; Yang et al. 2022).

Our second analysis takes into account that farming is a business and as such it pursues a profit, which makes the comparative assessment of CT and CA economic profitability foundational. In the absence of public subsidies for a transition to CA, economic profitability is pivotal in the decision of the farmer to adopt this land management (Smith et al. 2008a, 2008b). It also potentially provides for the risk aversion that characterizes farmers and induces conservativeness, preventing them from picking up new technologies (Aimin 2010). As reported by Boselli et al. (2020), yields results under CT and CA were comparable; so operational costs are key in determining economic profitability. In our analysis we report that there are different operational costs for CT and CA beyond that of tillage, such as the greater quantity of seeds required for sowing on mulch or the use of specific machinery for CA (table A1, Appendix A). On the whole, operational costs result lower for the CA management of plots. The comparison of the economic profitability of CT and CA is possible in this experiment as all the processes necessary for the implementation of CT or CA are purchased from contractors. Our analysis is therefore especially useful where these machines are available from contractors. The machines are mainly produced in South America, where CA has been applied since the 70 s and the relative industry is advanced. Regarding Europe, the industry is currently mainly present in Northern European countries where CA is implemented more extensively. In Italy, it is not yet widespread and experimenters reported availability of the CA specific machinery in approximately 20% of contractor accounts. With a wider diffusion of CA in Europe, also driven by the guidelines of the new CAP 2023, a parallel broader development can be foreseen for the specific CA machinery industry and subsequently their greater availability.

Finally, the composite efficiency analysis assesses the double profitability (environmental and economic) of CA vs CT, highlighting the potential of CA as a new business paradigm in the agricultural sector. In the DEA, the results on yields, CO_2_ sequestration and profit are studied in combination (as part of a single management practice), so as to identify the best management practice (BMP). Our results ascertain that, for the given temperate zone, the operational costs and the availability of the necessary specific machinery, CA is profitable, indeed more profitable than CT. Moreover, we can expect the distance (divergence) between the CT and the CA management practices in the DEA analysis to increase in time, as a result of an expected increase in soil fertility in CA-treated plots (Derpsch 2008; Lal 2015; Smith et al. 2008a, 2008b), and of a decrease in the operational costs consequent to a greater diffusion of specific CA machinery. The implications of this analysis are diverse. It allows the policy maker to assess that CA is concurrently environmentally performing and economically profitable, making it a win-win policy, particularly for the formulation of agricultural policies. In the EU, the Common Agricultural Policy (CAP) has recently been reformed (2023–2027 CAP), and among its objectives is a stronger contribution of agriculture to climate change mitigation and to the European Green Deal. Land is recognised as key for reaching a climate-neutral economy, thanks to its CO_2_ sequestration potential from the atmosphere, also considering the potential of Europe as a CO_2_ sink (CA could be implemented in 43% of the EU area) as confirmed by the EU Soil Strategy 2030 and the Carbon Farming Initiative. Consequently, the new CAP redirects part of its budget (of which 40% has to be climate relevant), also in the attempt to accelerate the transition to CA. This partly straightens out the inconsistency in the CAP related to the use of public money for the production of private goods (subsidies to production), and reshapes it into subsidies to the production of a public good (CO_2_ sequestration): “public money for public goods” (p. 297, Bateman and Balmford 2018). However, the related necessary definition of a certification of carbon removals is still an ongoing process, and attention is called to gather experience at local or regional level also to fine-tune methodologies and rules for monitoring, reporting and verifying the gains, or losses in the sequestered carbon.

The results of the DEA are also relevant to overcome the divergence between the social desirability of CA and its potential attractiveness to individual farmers (Knowler and Bradshaw 2007; Knowler 2015), since the economic and environmental convenience to adopt CA nudges the decision to implement it. Indeed, despite the general assumption that farmers’ decisions are mostly driven by economic rationality, costs are not the only important factor in the decision process for the adoption of agricultural conservation practices. The decision-making process regarding the adoption of new technologies is complex, and other factors are reported as equally important (Knowler and Bradshaw 2007; Lalani et al. 2016; Sattler and Nagel 2010). In fact, the measure that combines economic, social, and ecological requirements is assessed most favourable (Sattler and Nagel 2010). Accordingly, our DEA analysis highlights that CA is a BMP in economic and environmental terms, even before there are any subsidies for CO_2_ sequestration, which makes CA implementation susceptible to further risk reduction when subsidies become actual (as it is in the EU with the new 2023–2027 CAP).

The limitations of our paper are related to the site specificity of the field experiment data our analyses are based upon. These do not allow for a global validity of the results but, concurrently, respond to the request that emerges from the literature and that the new CAP has explicitly recognised, namely that there is no silver bullet that can be made to fit all circumstances (Kassam et al. 2014). This calls for a rigorous context-sensitive approach, also contributing to overcome the dogmatic and prescriptive attitude that is often reported (Giller et al. 2015). The analysis of the effects of other climate change mitigation startegies, such as afforestation and reforestation, agroforestry and other farming combining woody vegetation with croplands, targeted conversion of cropland to fallow or of set-aside areas to permanent grassland, restoration of peatlands and wetlands (European Commission 2021), are beyond the scope of this paper.

Regarding future research and, considering that CA advocates claim that the maximum benefit for the soil comes in the maintenance phase (Derpsch 2008; Lal 2002, 2015), assessing positive agro-ecosystem responses (i.e., efficient soil C and nutrient dynamics, as well as sustained crop yield) in the long run would be beneficial. This might be crucial in the light of the ambitious environmental objectives set by the EU in the “Farm to fork strategy” and the “Zero Pollution action plan”, such as to meet a 20% reduction in the use of fertilisers and/or a 50% abatement of nutrient losses by 2030 (Montanarella and Panagos 2021). Additionally, the impact on the diffusion of CA implementation and, the consequences on the production, availability, and prices of the dedicated machinery should be attested. In the context of a European Carbon market, to what extent the CO_2_ sequestration becomes part of the farmer’s profit function and the effect on the implementation of the CA as a carbon farming strategy will also be significant to study.

## Conclusions: what to do next

There are multiple available strategies for climate change mitigation, but this paper demonstrates that CA can be considered as a realistic and easy-to-use approach for boosting SOC sequestration in soils, thus mitigating climate change. However, despite its substantial potential, the transition to CA is not easy to implement. This is apparent if we think of the fragmentation of the world’s agricultural land. In the EU alone, the land devoted to agriculture is fragmented into 10.5 million agricultural holdings, two-thirds of which are less than 5 ha in size (EUROSTAT 2020), while in the US the number of farms in 2019 was estimated at 2,023,400, even though the average farm size is 180 ha (NASS 2020); not to mention developing countries, where the size of farms is around 1–1.5 hectare in Africa and even less in Asia. Moreover, particularly in developing countries, there is a lack of the necessary institutions to disseminate the CA-related know-how. Additionally, sociological, economic and political restraints are generally reported (Amundson and Biardeau 2018; Paustian et al. 2016; Pannell et al. 2006).

So what are the strategies and policies to be implemented to allow this virtuous transition to CA to come true? Implementing both top-down and bottom-up interventions will potentially achieve better and faster outcomes. To begin wi**t**h, as the new CAP has recognised, it is important to take action on the carbon credit market and introduce a price paid for sequestration, rather than just raising CO_2_ taxes. This would become a significant part of the farmers’ profit function and thus act as an incentive to transition to CA. Paying a price for CO_2_ sequestration would also have a welcome buffer effect on the volatility of agricultural profit, concurrently addressing the mentioned high risk aversion in farmers relative to the taking up of new production techniques (Aimin 2010; Liu et al. 2018; Lu et al. 2022). Preliminarily, the necessary technology for CO_2_ sequestration measurements needs to become standardised and systematic in order to guarantee space-time comparability.

In addition to the carbon credit market instrument aiming at triggering the transition bottom-up, the change must also be encouraged from above. This implies public support for the production of the public good that farmers provide while producing private goods. Furthermore, since CO_2_ sequestration is a global public good, developed countries could extend carbon credit markets and the relative payments outside their borders, comprising developing and least developed countries also possibly by partly redirecting and replacing existing heterogeneous aid measures and providing specific CA machinery. Finally, CA should additionally be implemented in urban areas, in order to counter the negative effect of heat islands (Heaviside et al. 2016) and pollution.

All in all, a change in the paradigm is needed and it is high time to encourage a conciliation of economic profitability and environmental performance so as to make ends meet between the “thinking of the end of the world” of scientists and scholars, and the “thinking of the end of the month” of agricultural entrepreneurs, the main actors in the called-for transition.

**Web sources**

https://ec.europa.eu/eurostat

https://www.nass.usda.gov/

### Supplementary information

APPENDIX A

## Footnotes

1 Total emissions from the agricultural sector have been growing exponentially since 1961, and now account for 18.4% of direct GHG emissions; rising to about a quarter when considering the food system as a whole. (Climate Watch, The World Resources Institute, 2022).

2 Intensive high-input agriculture characterised by large monocultures and high-yield productivity maintained through a strong reliance on specific practices.

3 The soil Carbon (C) pool is 3.3 times the size of the atmospheric C pool (760 Gt) (Lal, 2004; Paustian et al., 2016) therefore soil has the potential to offset fossil-fuel emissions by 0.4 to 1.2 Gt C/year, i.e., 5% to 15% of global emissions (Lal 2019), signalling that even small changes in the soil C stock can have strong impacts on the atmospheric concentration of CO_2_ (Lal 2020).

4 If the level of carbon stored by soils in the top 30–40 cm of soil increased by 0.4% (or 4‰) per year, the annual increase of carbon dioxide (CO_2_) in the atmosphere would be significantly reduced. https://4p1000.org/?lang=en.

5 However, Coelli et al. (2005) noticed that outcomes from both models are comparable, therefore, the choice of orientation is not crucial.

6 In particular for the combination of NT + hairy vetch.

7 By the time of writing, only 10 years to implement actions to combat climate change in order to keep the temperature increase at 1.5 °C (IPCC 2019).
