# Supplementary material for: Conservation Agriculture Impacts on Economic Profitability and Environmental Performance of Agroecosystems
Source: Environ Manage. 2023 Oct 16;73(3):532–45. doi: 10.1007/s00267-023-01874-1 (PMC10884138; doi:10.1007/s00267-023-01874-1)
Supplement: Supplementary file 1 — APPENDIX A [file 267_2023_1874_MOESM1_ESM.docx]

**APPENDIX A**

Table A1. CT and CA (NT+Cover Crop) Operational Costs

Source: Authors’ elaboration from Boselli et al. 2020

CT – Conventional Tillage

CA – Conservation Agriculture

NT – NoTill

CC – Cover Crop: Rye, Vetch, Mix

Table A2. Grain yield of winter wheat (2012, 2016, and 2019), maize (2013, 2014, 2017, and 2020),

and soybean (2015 and 2018), expressed as dry matter (Grain yield DM) and 14% grain moisture

(Grain yield 14%), as affected by tillage treatment (CT: conventional tillage; NT_rye:

no-till plus rye cover crop; NT_vetch: no-till plus hairy vetch cover crop; NT_mix: no-till plus the cover crop mixture).

*average values, as derived by the means of 4 randomized replicates

Source: Boselli et al., 2020

Table A3. Mean EXW Prices €⁄ t per Crop 2011-2020, Bologna Chamber of Commerce

Source: Authors’ calculations from the Bologna Chamber of Commerce cereals and legumes price list

Table A4. Mean and Standard Deviation for SOC per Crop and per System (CT CA)

| Crop System | CT  Mean | CT  Std Deviation | NoTill  Mean | NoTill  Std Deviation |
| --- | --- | --- | --- | --- |
| Wheat | 51.19 | 1.86 | 58.31 | 6.24 |
| Soybean | 50.80 | 1.42 | 60.35 | 3.96 |
| Maize | 51.16 | 1.39 | 60.25 | 4.85 |
| SOC_Total | 51.09 | 1.53 | 59.62 | 5.23 |

Source: Authors’ calculations

CT : conventional tillage

Table A5. Mean and standard deviation for yield per crop and per system (CT CA)

| Crop System | CT  Mean | CT  Standard Deviation | Notill  Mean | Notill  Standard Deviation |
| --- | --- | --- | --- | --- |
| Wheat | 7.503 | 1.127 | 7.496 | 1.318 |
| Soybean | 3.210 | 0.127 | 3.502 | 0.301 |
| Maize | 12.470 | 1.417 | 12.347 | 0.974 |
| Yield_Total | 8.757 | 4.031 | 8.764 | 3.718 |

Source: Authors’ calculations

CT : conventional tillage

Table A6. Descriptive Statistics For Costs Per Crop, Per Year, Per System (CT CA)

|  | CT | NT_Rye | NT_Vetch | NT_Mix | Mean_ NT |
| --- | --- | --- | --- | --- | --- |
| Min | 1305.6 | 861.6 | 861.6 | 861.6 | 861.6 |
| Max | 1872.13 | 1583.13 | 1690.13 | 1648.13 | 1640.46 |
| Mean | 1569.04 | 1227.26 | 1298.59 | 1270.59 | 1265.48 |
| Median | 1484.05 | 1196.05 | 1303.05 | 1261.05 | 1253.38 |
| Std. Dev. | 231.1 | 291.16 | 338.11 | 319.43 | 316.06 |
| Assymetry | 0.13 | -0.07 | -0.18 | -0.14 | -0.13 |
| Curtosis | -2.01 | -1.94 | -1.9 | -1.91 | -1.92 |
| n obs | 9 | 9 | 9 | 9 | 9 |

Source: Authors’ calculations

CT : conventional tillage

CA : NT_rye: no-till plus rye cover crop; NT_vetch: no-till plus hairy vetch cover crop; NT_mix: no-till plus the cover crop mixture
